# Boron nitride encapsulated copper nanoparticles: a facile one-step synthesis and their effect on thermal decomposition of ammonium perchlorate

**DOI:** 10.1038/srep16736

**Published:** 2015-11-16

**Authors:** Caijin Huang, Qiuwen liu, Wenjie Fan, Xiaoqing Qiu

**Affiliations:** 1 State Key Laboratory of Photocatalysis on Energy and Environment, College of Chemistry, Fuzhou University, Fuzhou 350002, P. R. China

## Abstract

Reactivity is of great importance for metal nanoparticles used as catalysts, biomaterials and advanced sensors, but seeking for high reactivity seems to be conflict with high chemical stability required for metal nanoparticles. There is a subtle balance between reactivity and stability. This could be reached for colloidal metal nanoparticles using organic capping reagents, whereas it is challenging for powder metal nanoparticles. Here, we developed an alternative approach to encapsulate copper nanoparticles with a chemical inertness material—hexagonal boron nitride. The wrapped copper nanoparticles not only exhibit high oxidation resistance under air atmosphere, but also keep excellent promoting effect on thermal decomposition of ammonium perchlorate. This approach opens the way to design metal nanoparticles with both high stability and reactivity for nanocatalysts and their technological application.

Metal nanoparticles have been widely used in various fields including electronics, photonics, biomedicine, and chemistry because of their fascinating properties^1^,^2^,^3^,^4^,^5^,^6^. Most of investigations on metal nanoparticles focus on their size, shape and composition rather than their stability and reactivity^7^,^8^,^9^,^10^. However, both stability and reactivity are two critical factors for metal nanoparticles to ensure their subsequent characterization, functional formulation and further application. While the dimensions of metal materials tend to be at the nanometric scale, they become more reactive and unstable because of their high surface energy and large surface-to-volume ratio^11^. As a result, stability and reactivity seem to be contradictive and difficult to be simultaneously attained in view of surface chemistry and process^12^,^13^,^14^. Therefore, synthesizing metal nanoparticles with both high stability and robust reactivity remains challenging and desired.

Among metal nanoparticles, copper (Cu) nanoparticles attract great interest due to their outstanding and distinct features in catalysis, electronics and photonics^4^,^5^,^6^. However, Cu nanoparticles are very easy to be oxidized under air condition to form Cu oxides or hydroxides outside themselves influencing/changing their chemical and physical properties (e.g. catalytic activity, conductivity)^15^. To avoid oxidation, the surfactants or organic capping agents for anti–agglomeration used in the preparation process can prevent the obtained products from being oxidized and be stored in solution for long^16^. However, as for powder Cu nanoparticles, the organic surfactants are not enough stable in air for long or under high-temperature O_2_-rich condition required in many industrially catalytic processes^15^. From the viewpoint of materials science and technological application, wrapping air-sensitive metal nanoparticles with a protective material would be an alternative and effective strategy. Inspiringly, several such materials have been recently found, such as SiO_2_, Al_2_O_3_, carbon and boron nitride^17^,^18^,^19^,^20^,^21^,^22^. Moreover, some wrapped metal nanoparticles not only acquire remarkable stability but also strongly promote physicochemical performances^23^,^24^, which is so intriguing and highly motivates one to extend the breadth of practical applications of nanoparticles. For example, Joo *et al*. found that Pt/mesoporous silica core-shell nanoparticles remained much stable at high temperature and simultaneously presented high catalytic activity of CO oxidation^25^. Wan and co-workers demonstrated that carbon-encapsulated tin nanoparticles displayed high specific capacity and excellent cycling performance, making them a promising anode materials in lithium-ions batteries^26^. Recently, hexagonal boron nitride (*h*-BN) has been successfully used in our previous studies as an excellent plane-like support for dispersing and stabilizing noble metal and Cu oxide catalysts^27^,^28^. *h*-BN is a structural analogue of graphite with remarkable properties such as chemical inertness, thermal stability, thermal conductivity and electric insulation. Its high stability and resistance to oxidation enable it an ideal candidate as an encapsulating material.

Herein, we applied *h*-BN as a protective and dispersing substrate to wrap Cu nanoparticles by one-step pyrolytic decomposition of a mixture of cupric salts, boron oxide and urea. The thermal decomposition results in the formation of both *h*-BN and Cu nanoparticles. The formed Cu nanoparticles are highly dispersed and encapsulated within layered *h*-BN sheets. To evaluate the reactivity of the as-prepared *h*-BN-wrapped Cu nanoparticles (noted as Cu@*h*-BN in this paper), we investigate their effects on thermal decomposition of ammonium perchlorate (AP). AP is an oxidizer commonly used as composite solid propellants for rockets and missiles. The combustion of AP is generally promoted by transition metal oxides or bulk metal powder^29^,^30^,^31^. We found that the Cu@*h*-BN composites are much stable in air for long and show high activity for the thermal decomposition and heat release of AP.

## Results

**Figure 1:**
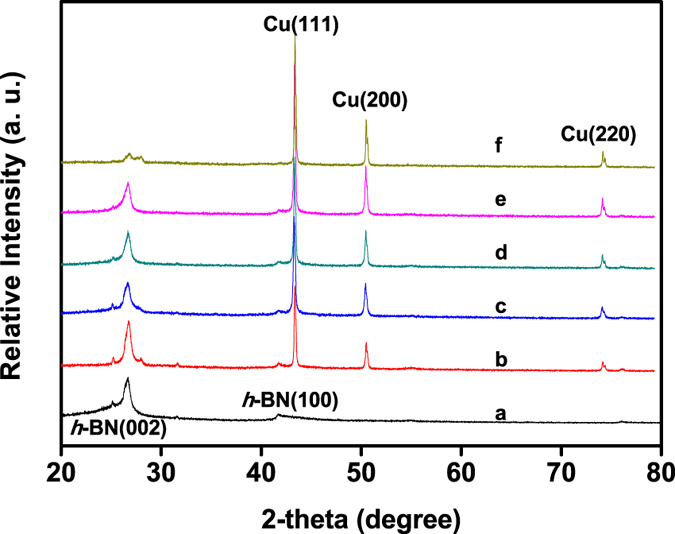
XRD patterns of the as-synthesized Cu@*h*-BN samples stored in air for three months, (**a**) pure *h*-BN, (**b**) 10.0 wt% Cu@*h*-BN, (**c**) 14.2 wt% Cu@*h*-BN, (**d**) 18.1 wt% Cu@*h*-BN, (**e**) 25.0 wt% Cu@*h*-BN, (**f**) 30.7 wt% Cu@*h*-BN, respectively.

**Figure 2:**
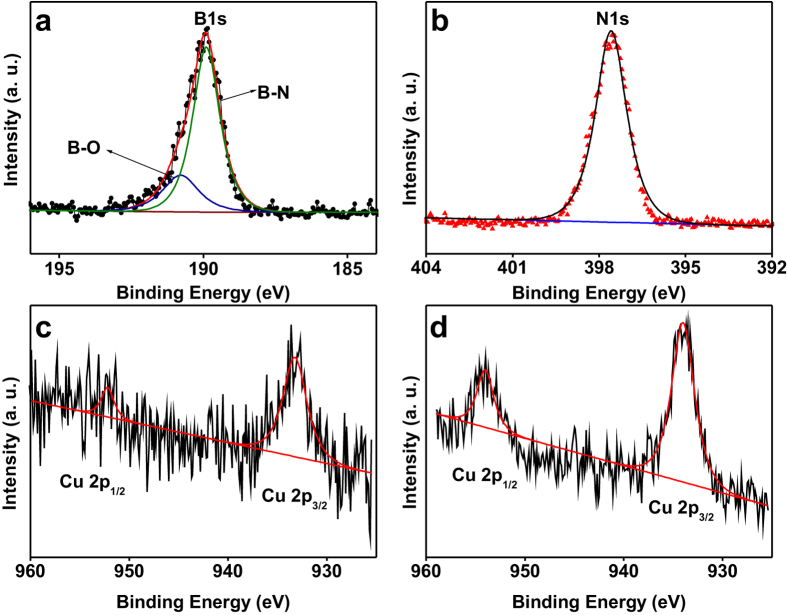
XPS spectra of samples. (**a**) B 1s, (**b**) N 1s, and (**c**) Cu 2p core-level spectra of the 25.0 wt% Cu@*h*-BN sample, (**d**) Cu 2p core-level spectrum of the 30.7 wt% Cu@*h*-BN sample.

Cu nanoparticles encapsulated by *h*-BN were synthesized via one step thermal decomposition of the mixture of boron oxide, urea and cupric nitrate. Figure 1 shows the powder X-ray diffraction (XRD) patterns of the samples stored in air for three months. By comparing diffraction peaks with the standard PDF cards (JCPDS card No. 85–1068 and No. 04–0836), the phase structures of *h*-BN and Cu can be resolved. The peaks located at 26.7° and 41.7° represent the characteristic reflections (002) and (100) of *h*-BN, respectively. The diffraction peaks at 43.4°, 50.5° and 74.1° correspond to the (111), (200) and (220) planes of Cu, respectively. For comparison, the XRD patterns of the freshly prepared samples are given in Fig. S1. No clear changes have been found for the diffractions from Cu between the freshly prepared samples and the samples stored for three months. Moreover, it could be found that the XRD signals for Cu gradually increase with its content increasing in the samples, which is very similar to those of Cu-Ni-Al-Co-Cr-Fe-Si alloy systems^8^. The diffraction intensity of *h*-BN is weaker and broader than those of Cu due to its low crystallinity. Note that no other peaks related to Cu oxides are observed under the detection limit and sensibility of the XRD apparatus, which can also be confirmed by the following Raman (Fig. S3) and X-ray photoelectron spectroscopy (XPS) (Fig. 2) analyses. In addition, there are two small peaks occurred at 25.3° and 31.7°, which can be attributed to the formation of NH_4_B_5_O_8_·4H_2_O (JCPDS card No. 31− 0043), as well as one small peak at 27.8° originating from B_2_O_3_ (JCPDS card No. 06–0297). This is likely due to the presence of O_2_, moisture and trace ammonium gas retained within *h*-BN layers.

In order to get further information on physics and chemistry of our samples, the diffuse reflectance spectroscopy (DRS) has been collected. As shown in Fig. S2, there is a broad peak centered at *ca*. 570 nm for the Cu@*h*-BN samples, which is attributed to the localized surface plasmon of Cu nanoparticles^32^,^33^. Additionally, a much slight and broad absorption band of *h*-BN can be observed over the visible regime due to the doping of oxygen and carbon in the *h*-BN framework^34^. Raman spectra of samples were obtained using a 532 nm laser source. As shown in Fig. S3, the Raman peak of *h*-BN is located at 1367 cm^−1^, which can be assigned to the typical B-N stretching vibration mode (E_2g_)^35^. Note that there are not any obvious peaks at 220 cm^−1^ and 295 cm^−1^ ascribed to Cu oxides^27^,^36^, as for Cu@*h*-BN samples. However, the incorporation of Cu can induce a significant shift of the vibration frequencies of the B-N, suggesting the existence of a strong interaction between the *h*-BN and Cu nanoparticles in the Cu@*h*-BN composites. The peaks of B-N stretching vibration are located at 1340 and 1355 cm^−1^, presenting 28 and 13 cm^−1^ downshift for 25.0 wt% Cu@*h*-BN and 30.7 wt% Cu@*h*-BN samples, respectively. Generally, shifting in E_2g_ can occur under different strain conditions within layers^35^. Similar phenomena have been observed on the work of graphene by Calizo^37^ and Ferrari^38^. The strong interaction between *h*-BN and Cu nanoparticles might be beneficial for the stability and reactivity of the Cu component.

XPS was further employed to get detailed information of the chemical composition and element states of the samples. Figure 2 and S4a shows the XPS spectra of the samples 25.0 wt% Cu@*h*-BN and 30.7 wt% Cu@*h*-BN. As illustrated in Fig. 2a, the main peak at 189.9 eV with a shoulder at 190.8 eV in B 1s spectra can be assigned to B-N bonds^39^ and B-O bonds, respectively^27^,^28^. The N 1s core-level XPS spectrum (Fig. 2b) shows a strong photoelectron signal at 397.6 eV, which can be assigned to the B-N bonds, consistent with literature values for N^3−^ in BN layers^40^,^41^. In the case of the Cu 2p core-level spectrum (Fig. 2c,d), two intense peaks located at 933.9 and 952.5 eV are observed, which can be designated as Cu 2p_3/2_ and Cu 2p_1/2_ spin-orbital components^42^, respectively. Importantly, no satellite peaks corresponding to Cu^2+^ species are detected^43^,^44^, ruling out the CuO in our samples. Moreover, the Cu LMM Auger peak is observed at kinetic energy of 918 eV (Fig. S4b), similar to that found for pure copper metal^43^,^44^. Thus, the XPS results in combination with the above XRD, Raman analyses demonstrate that Cu nanoparticles can be stabilized in air and not be oxidized by the protection of *h*-BN wrapping.

**Figure 3:**
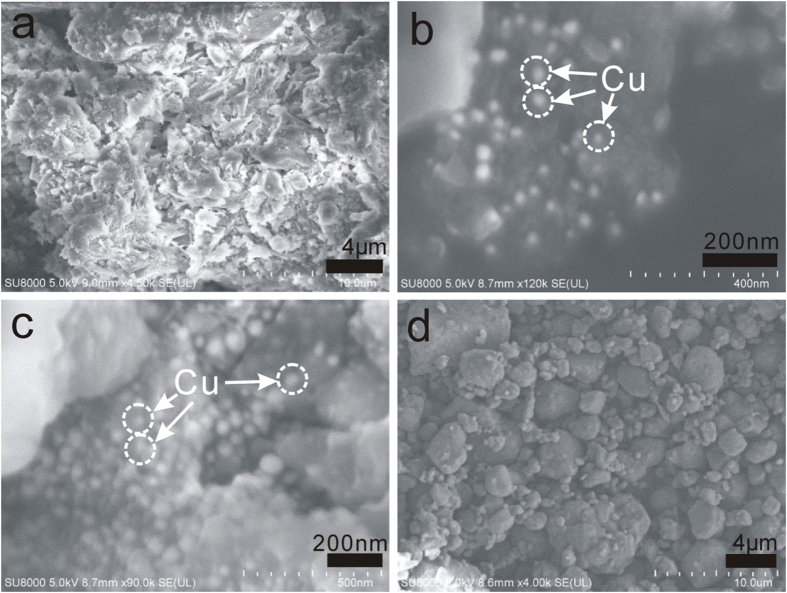
SEM images of (**a**) pure *h*-BN, (**b**) 25.0 wt% Cu@*h*-BN, (**c**) 30.7 wt% Cu@*h*-BN and (**d**) pure AP.

**Figure 4:**
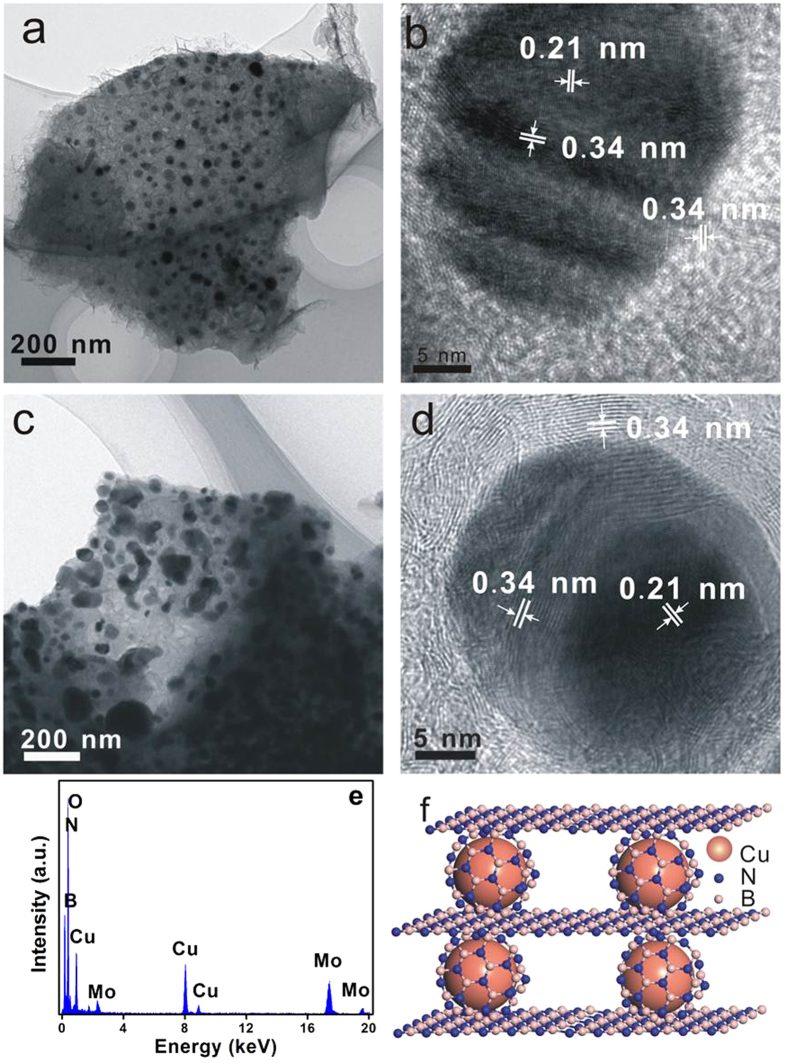
TEM and HRTEM images of (**a,b**) 25.0 wt% Cu@*h*-BN and (**c,d**) 30.7 wt% Cu@*h*-BN. (**e**) EDX of the sample 25.0 wt% Cu@*h*-BN. (**f**) Schematic achitecture of Cu@*h*-BN.

The morphology, architecture and microstructures of the samples were next investigated using field-emission scanning (SEM) and transmission electron microscopy (TEM) obsvations. Figure 3 shows the SEM images of the Cu@*h*-BN samples with various Cu contents and commercial AP powder. As displayed in the Fig. 3a, the pure h-BN features a layered sturecture. After incorporation with Cu, the Cu particles can be easily distingusihed with a highly contrasty image due to the high electric resistivity of *h*-BN. As shown in Fig. 3b,c, the Cu nanoparticles are higly dispersed with a diameter of 40–70 nm, and coated by *h*-BN thin sheets. Moreover, the population of nanoparticles can be controlled by the increasing the Cu contents in the Cu@*h*-BN composites. Consistent with the SEM results, the TEM images show the Cu@*h*-BN composites are composed of spherical nanoparticles and thin sheets (Fig. 4a,c). Moreover, from their corresponding high-resolution TEM (HR-TEM) images (Fig. 4b,d), there are two crystal lattice spacing of 0.34 and 0.21 nm coresponding to the (002) crystal plane of *h*-BN^28^ and the (111) crystal face of Cu nanoparticles^45^, respectively. Furthermore, it can be seen that Cu nanoparticles are also surrounded by *h*-BN sheets, which are in good agreement with the SEM observation. In addition, surface analysis using energy dispersive X-ray (EDX) presented in Fig. 4e demonstrates that the sample consists of B, N, O and Cu, which is in agreement with the XPS spectrum shown in Fig. S4a. The element Mo comes from the Mo support used in TEM measurement. Therefore, it can be inferred that the architecture of Cu nanoparticles is under the support and encapsulation of *h*-BN , which is schematically illuminated in Fig. 4f.

**Figure 5:**
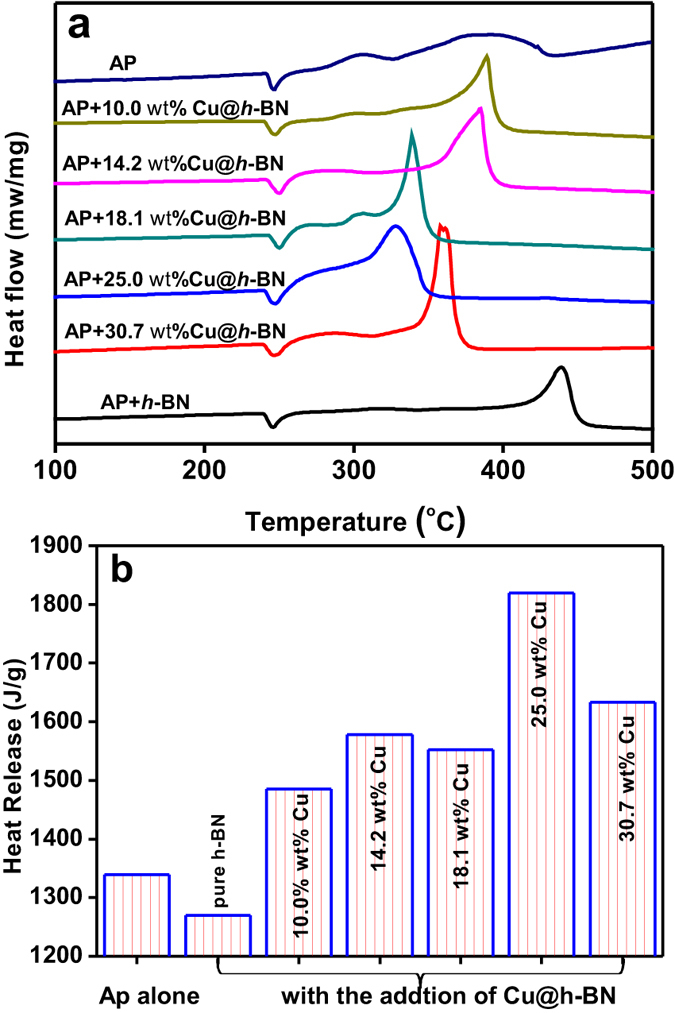
(**a**) DTA of AP decomposition in the presence of the as-obtained samples, (**b**) heat release during the exothermic process occurred in (**a**).

Following the above analysis, the Cu@*h*-BN composites with various Cu contents are initially used as the additives to promote the thermal decomposition of AP with and attempt to study the the reactivity of the Cu nanoparticles stabilized by *h*-BN. Since the thermal decompostion of AP can be greatly influenced by the sizes and morphologies of AP^46^, the SEM observation of the AP was conducted. As shown in Fig. 3d, the AP exhibits a inhomogeneity in sizes ranging from hundreds nanometer to about 10 um. Differential thermal analysis (DTA) and heat release analyses have been carried out to study the promoting effects of the Cu@*h*-BN composites on the thermodynamic behavior of the AP (the samples Cu@*h*-BN: AP = 2: 98 wt/wt) during the reaction at a heating rate of 10 °C/min. As for AP alone (Fig. 5a), one endothermic peak at 246 °C is observed, which comes from the phase transition from orthorhombic to cubic form^46^, while two exothermic peaks located at the range of 280 to 450 °C orginate from the AP decomposition. The small exothermic peak at 307 °C corresponds to low temperature decomposition resulting from the partial decomposition of AP, and another broad peak at 391 °C is attributed to the complete decomposition^47^. The decomposition of AP seems to be hindered by the addition of pure h-BN, implying the *h*-BN can not act as a good promoter for the AP decomposition. This is probably due to the electric insulating of *h*-BN disfavoring charge transfer occurred during the AP decomposition process^46^. Encouragingly, with the addition of the Cu@*h*-BN samples, the exothermic peaks for the complete decomposition of AP become sharper and shift to lower temperature, despite of no significant changes on the phase transition of AP. This implies that our Cu@*h*-BN samples can trigger complete decomposition at lower temperature and promote its decomposition process. Moreover, the remarkable decrease of complete decomposition temperature leads to that the two exothermic peaks tend to be integrated into an exothermic peak, which is beneficial for the heat release of AP and its practical application as the propellants. The promoting effect of Cu@*h*-BN samples first increase with the Cu content up to 25.0 wt%, and decrease with further increasing the Cu content to 30.7 wt%. Among all the samples, Cu@*h*-BN sample with 25.0 wt% is found to be most active. The promoting effect could be explained by that the decomposition products NH_3_ and HClO_4_ are absorbed on the additive surface and initiate their subsequent redox reaction^47^, which will be discussed below. Moreover, we also investigated heat release for AP decomposition to get more details on the promoting effects of Cu@*h*-BN samples. Figure 5b demonstrates that the presence of the Cu@*h*-BN samples can lead to much more heat release than AP alone. the overall heat releases are determined to be 1339, 1270, 1485, 1578, 1552, 1820 and 1633 J/g for AP alone and AP with the addition of Cu@*h*-BN samples, respectively, demonstrating that the decomposition of AP is advanced by the Cu@*h*-BN samples, especially by Cu@*h*-BN with 25.0 wt% Cu content (1820 J/g). Moreover, we have compared the activity of fresh Cu@*h*-BN samples with 25.0 wt% Cu content and the one stored in air atmosphere for three months. From Fig. S6, the similar DTA curves are observed for the fresh sample and the stored one, further confirming the high stability of Cu@*h*-BN samples.

**Figure 6:**
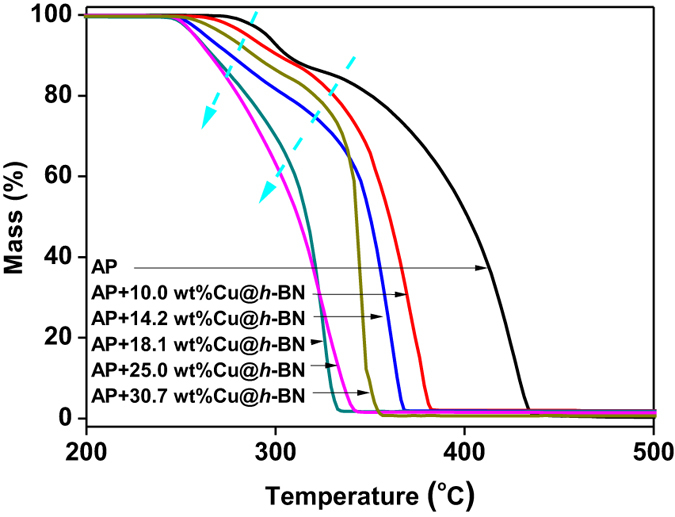
(**a**) TG of the mixture of AP and the Cu@*h*-BN with different Cu contents.

The decomposition of AP is further investigated by thermogravimetric analysis (TGA). As shown in Fig. 6, the TGA weight loss curve of AP exhibits two clear steps. One is about 10% in the range of 270–310 °C, and the other is about 90% weight loss from 310–440 °C, corresponding to the low-temperature and high-temperature decompositions, respectively. With the addition of Cu@*h*-BN samples, it is found that the decomposition of AP moves to lower temperature. What is more, the low-temperature and high-temperature decompositions of AP seem to be merged into one process with the increasing the Cu content in the additives of Cu@*h*-BN. It should be mentioned that the TGA weight loss curves of AP assisted with Cu@*h*-BN with 25.0 and 18.1 wt% Cu content are closed. The curves of the Cu@*h*-BN 18.1 wt% Cu content is even lower than that of the Cu@*h*-BN 25.0 wt% Cu content in the range of the ending minor AP weigh loss. The ending minor weigh loss should not the key contribution of the heat release of AP decomposition. Since the promoting effect of the additives for AP decomposition is generally evaluated based on the summit peak of the decomposition and the heat release^8^,^30^,^47^, the sample of Cu@*h*-BN with 25.0 wt% Cu content is regarded as the most active one, and taken as example for the further investigation in this study.

## Discussion

**Figure 7:**
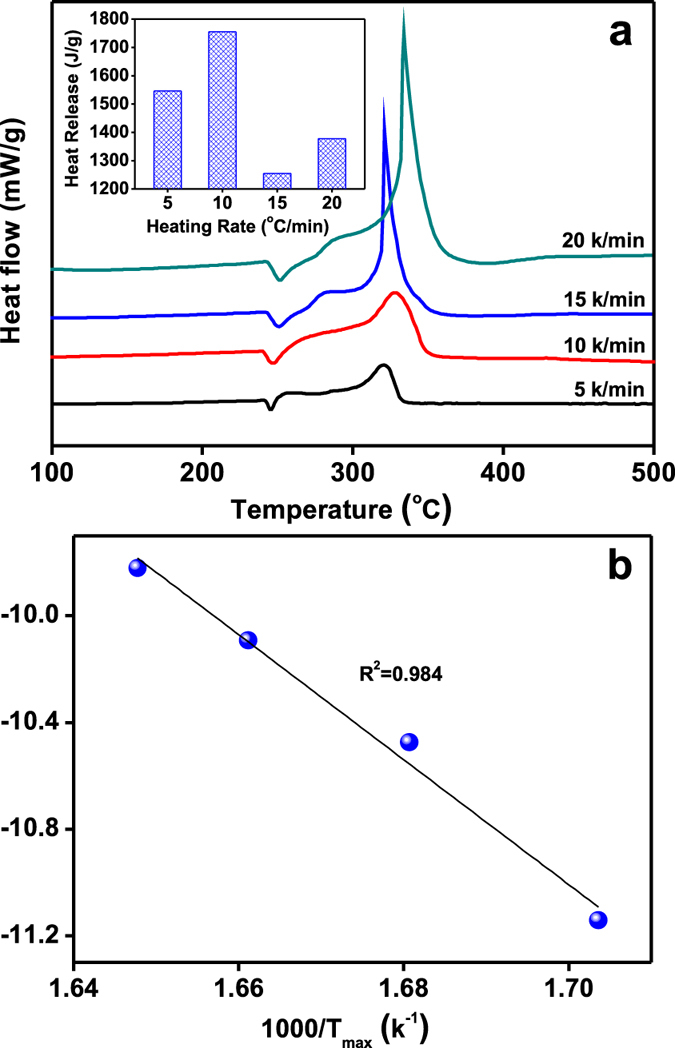
(**a**) DTA curves of the mixture of AP and 25.0 wt% Cu@*h*-BN (mass ratio is 98:2) at different heat rates, (inset) corresponding heat release during the exothermic process, (**b**) ln (β/T^2^_max_) as a function of (1/T_max_). β and T_max_ are the heating rate and the related summit peak temperature presented in (**a**), respectively.

To study the effects of the dosage of Cu@*h*-BN samples on the thermal decomposition behaviors of AP, the 25.0 wt% Cu@*h*-BN samples and AP was pre-mixed with a mass ratio ranging from 1:99 to 10:90 to prepare the target mixtures. The decomposition of the mixtures was carried out at a heating rate of 10 °C/min in a N_2_ atmosphere. As shown in Fig. S5a, the amount of our samples has no evident impacts on the exothermic peak. By comparing the related heat release (Fig. S5b), it can be found that the best mass ratio of Cu@*h*-BN samples and AP should be 2: 98. Moreover, it is reported that the heat release of AP is highly dependent on the heating rates^48^. Figure 7a shows the DTA curves of the mixtrues of AP and 25.0 wt % Cu@*h*-BN at different heating rates, *i.e*. 5, 10, 15, and 20 °C/min. It can be seen that the heating rate has an obvious effect on the intensity and area of the exothermic peak. From the inset of Fig. 7a, the highest heat release is achieved under the heating rate of 10 °C/min. Most importantly, the kinetic parameter of activation energy (*E*_*a*_) for AP decomposition with Cu@*h*-BN samples can be derived from the exothermic peak temperature dependence as a function of heating rate. According to Kissinger’s method^49^, the *E*_*a*_ can be written as:


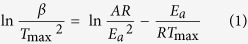


where *β, T*_*max*_*, R*, and *A* represent the heating rate in °C/min, the summit peak temperature, the ideal gas constant and the pre exponential factor, respectively. The summit peak temperature T_max_ can be obtained from Fig. 7a. According to the above equation, *E*_*a*_ is determined to be 197 kJ/mol, from the slope of the linear portion in the plot of ln (β/T^2^_max_) vs (1/T_max_) (shown in Fig. 7b). This value is about 130 kJ/mol lower than that for AP alone^50^, indicating that the presence of the Cu@*h*-BN samples can lower the required reaction temperature and facilitate the decomposition reaction.

**Figure 8:**
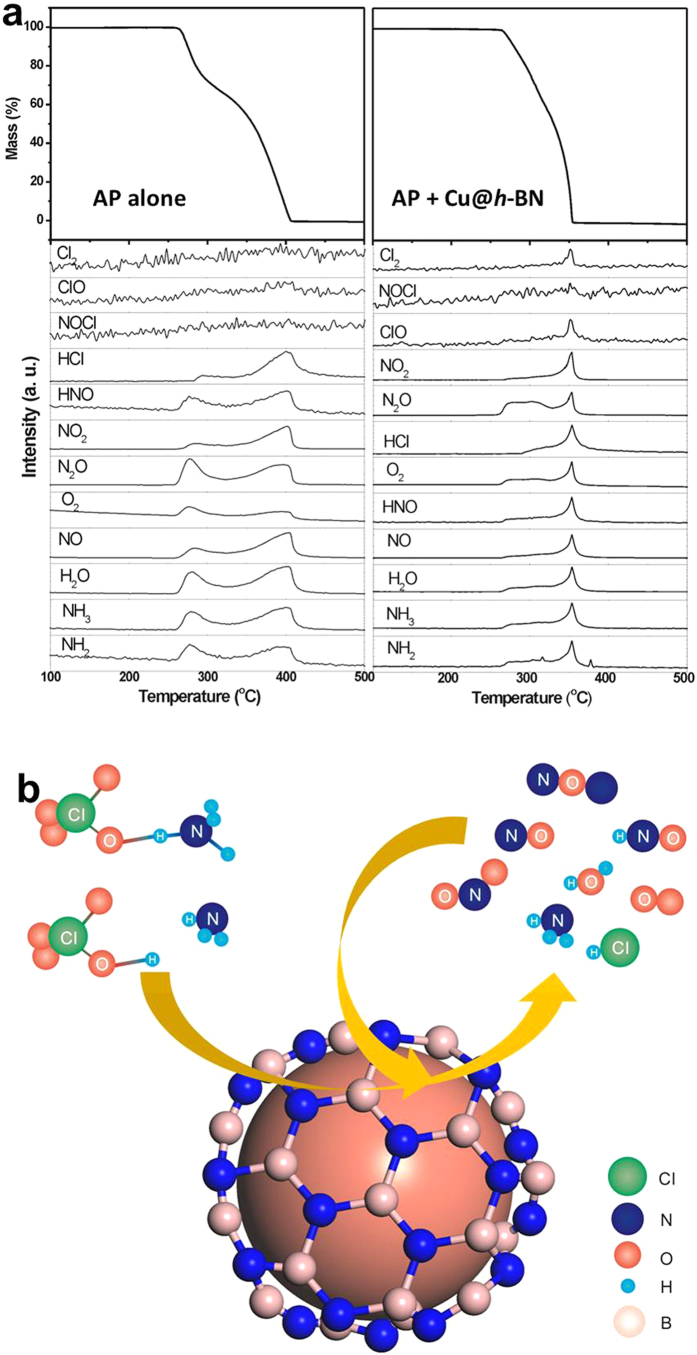
(**a**) TG-MS analyses of thermal decoposition of pure AP (left) and the mixture of AP and 25.0 wt% Cu@*h*-BN (mass ratio is 98:2) at a heating rate of 10 °C/min, (**b**) Schematic for Cu@*h*-BN catalyzing AP thermal decomposition reaction.

To get more information about the pyrolytic (intermediate) products of AP, thermogravimetry coupled with mass spectroscopy (TG-MS) measurement was conducted for *in-situ* analysis of the reaction products and discussing the related possible mechanism. For pure AP decomposition (Fig. 8a), NH_3_, NH_2_, NO, N_2_O, NO_2_, HNO, H_2_O, O_2_, HCl and trace amount of Cl_2_ and ClO, can be found at low and high temperature, which are also reported in the literature^51^. While adding the additives, these products increase dramatically from low-temperature decomposition stage to high-temperature process, especially for the products Cl_2_ and ClO (Fig. 8a). The promoting effect of Cu@*h*-BN is therefore more significant on the high-temperature decomposition process than on the low-temperature decomposition, which can also be confirmed by DTA and TG data presented in Figs 5 and 6. Actually, the thermal decomposition of AP is essentially complex, because the compound AP is composed of four elements and the decomposition process is related to the full oxidation of nitrogen and chlorine^48^. There are several points of view on the mechanism of thermal decomposition of AP. Bircomshaw and Newman suggested that decomposition process is driven by electron transfer from perchlorate ion to ammonium ion^46^. Additionally, the mechanism based on electron transition within energy bands was proposed by Raevsky and Manelis^46^. However, since AP is a dielectric with a band gap of 5.6 eV, there is a low probability for both electron transfer and band-to-band transition during the decomposition process^46^. Instead, Boldyrev^46^ presented a favorable mechanism based on proton transfer from the cation NH_4_^+^ to the anion ClO_4_^−^, which initiates and sustains the decomposition reaction. For our study, the process of low-temperature decomposition is considered to start with the formation of nuclei inside AP^52^, which is hardly affected by the presence of Cu@h-BN samples. In other word, the additives could not easily get involved in the proton transfer process at low-temperature stage. This can be confirmed by heat release presented in Fig. 5a. In contrast, at high temperature, the complete decomposition reaction takes place on the surface of AP covered/contacted with the Cu@*h*-BN samples^47^. Additionally, the decomposed and sublimated molecules of NH_3_ and HClO_4_ react either in the gas phase or in the adsorbed layer on the surface of Cu@*h*-BN samples^52^. On the other hand, the side products (such as NO, N_2_O, H_2_O, O_2_) can trigger secondary chemical reactions in the gas phase or on the surface of Cu@*h*-BN samples, too^52^. The reactions can schematically depicted in Fig. 8b. To test this hypothesis, we checked the XRD patterns of the samples after AP decomposition (Fig. S7). It is found that the peaks of Cu metal decrease accompanying the appearance of CuO. This finding is reasonable, because the strong oxidizing molecules, such as O_2_, Cl_2_ and ClO, were produced over Cu@*h*-BN samples during the decomposition of AP under high temperature. The experiment results also indicates that the Cu nanoparticles can be stabilized by *h*-BN sheets against the oxidiation in air for long time, but Cu nanoparticles are not inert and can perform a good promoting effect for AP decomposition. In these reactions, Cu nanoparticles may enhance the heterogeneous redox reactions by promoting charge mobility and transfer rate through the electrically conductive metal surface or the formal oxidation/reduction states transformation^52^ at the high-temperature decomposition stage.

In summary, we successfully developed a feasible strategy to synthesize Cu nanoparticles covered and highly dispersed by *h*-BN layers via one-step thermal decomposition. The Cu nanoparticles remain very stable in air at room temperature without any obvious oxidation. Furthermore, high anti-oxidation Cu nanoparticles exhibit remarkable promoting activity for the thermal decomposition of AP, indicating the coating of *h*-BN layers does not impede catalytic reactions occurred on the metal surface. The encapsulation by *h*-BN thus not only stabilizes Cu nanoparticles but also maintains their high activity. This approach can potentially be extended to other metal nanoparticles. The present study holds great promise for the formulations of powder metal nanoparticles used for solid-solid or solid-gas heterocatalysis and for practical applications in advanced nanosized electronic devices.

## Methods

### Preparation of *h*-BN encapsulated Cu nanoparticles

Typically, 3 g B_2_O_3_, 6 g urea and 0.9–3.6 g Cu(NO_3_)_2_ were homogenously mixed and then heated in a tube furnace at 1250 °C in ammonium gas for 5 hours, followed by a cool to the room temperature. According to the addition amount x of Cu(NO_3_)_2_. For comparison, pure *h*-BN was also synthesized using starting reagents B_2_O_3_ and urea. The as-prepared samples were stored in air at room temperature.

### Characterization of the as-prepared samples

XRD patterns of the samples were carried out on a Bruker D8 Advance diffractometer with Cu Kα1 radiation at room temperature. The UV/Vis diffuse reflectance spectra were acquired on a Varian Cary 500 Scan UV/Vis system. Raman spectra were recorded from 500 to 2500 cm^−1^ on a Renishaw inVia Raman Microphrobe at 532 nm laser excitation. The XPS was analyzed on a SHIMADZU (Amicus), using Al Kα X-ray as an excitation source (1486.8 eV). The morphologies and microstructures of the samples were explored by field emission scanning electron microscopy on a Hitachi New Generation SU8100 apparatus and transmission electron microscopy on a TECNAI F30 instrument operated at 200 kV.

### Reactivity test

the reactivity of the Cu@*h*-BN sample was evaluated by thermal decomposition of AP in a covered crucible under Nitrogen atmosphere. To investigate the dependence of amount of Cu@*h*-BN sample on AP decomposition, AP and Cu@*h*-BN sample were pre-mixed at a mass ratio ranging from 99: 1 to 90: 10 to prepare the target samples. The activation energies of AP decomposition with Cu@*h*-BN sample was explored by varying the heating rates. Thermal gravity (TG) was performed on NETZSCH STA 449 F3 thermal analyzer working from room temperature to 500 °C in an N_2_ atmosphere. The thermal behaviors of the mixtures were carried out on NETZSCH DTA 404 PC Differential thermal analyzer (DTA) at different heating rate from room temperature to 500 °C in an N_2_ atmosphere. Mass spectrometric data were obtained on the NETZSCH STA449C-QMS403C thermal gravity-mass spectrometry (TG-MS).

## Additional Information

**How to cite this article**: Huang, C. *et al*. Boron nitride encapsulated copper nanoparticles: a facile one-step synthesis and their effect on thermal decomposition of ammonium perchlorate. *Sci. Rep*. **5**, 16736; doi: 10.1038/srep16736 (2015).

## Supplementary Material

Supplementary Information
