# Supplementary material for: Boron nitride encapsulated copper nanoparticles: a facile one-step synthesis and their effect on thermal decomposition of ammonium perchlorate
Source: Sci Rep. 2015 Nov 16;5:16736. doi: 10.1038/srep16736 (PMC4645124; doi:10.1038/srep16736)
Supplement: Supplementary Information [file srep16736-s1.doc]

Boron nitride encapsulated Cu nanoparticles: a facile one-step synthesis and their effect on thermal decomposition of ammonium perchlorate

*Caijin Huang, Qiuwen liu, Wenjie Fan, and Xiaoqing Qiu^*^*

State Key Laboratory of Photocatalysis on Energy and Environment, College of Chemistry, Fuzhou University, Fuzhou 350002, P. R. China.

**Figure S1.** XRD patterns of the freshly synthesized Cu@*h*-BN with various copper content, (a) 10.0 wt% Cu content, (b) 14.2 wt% Cu content, (c) 18.1 wt% Cu content, (d) 25.0 wt% Cu content, (e) 30.7 wt% Cu content, respectively.


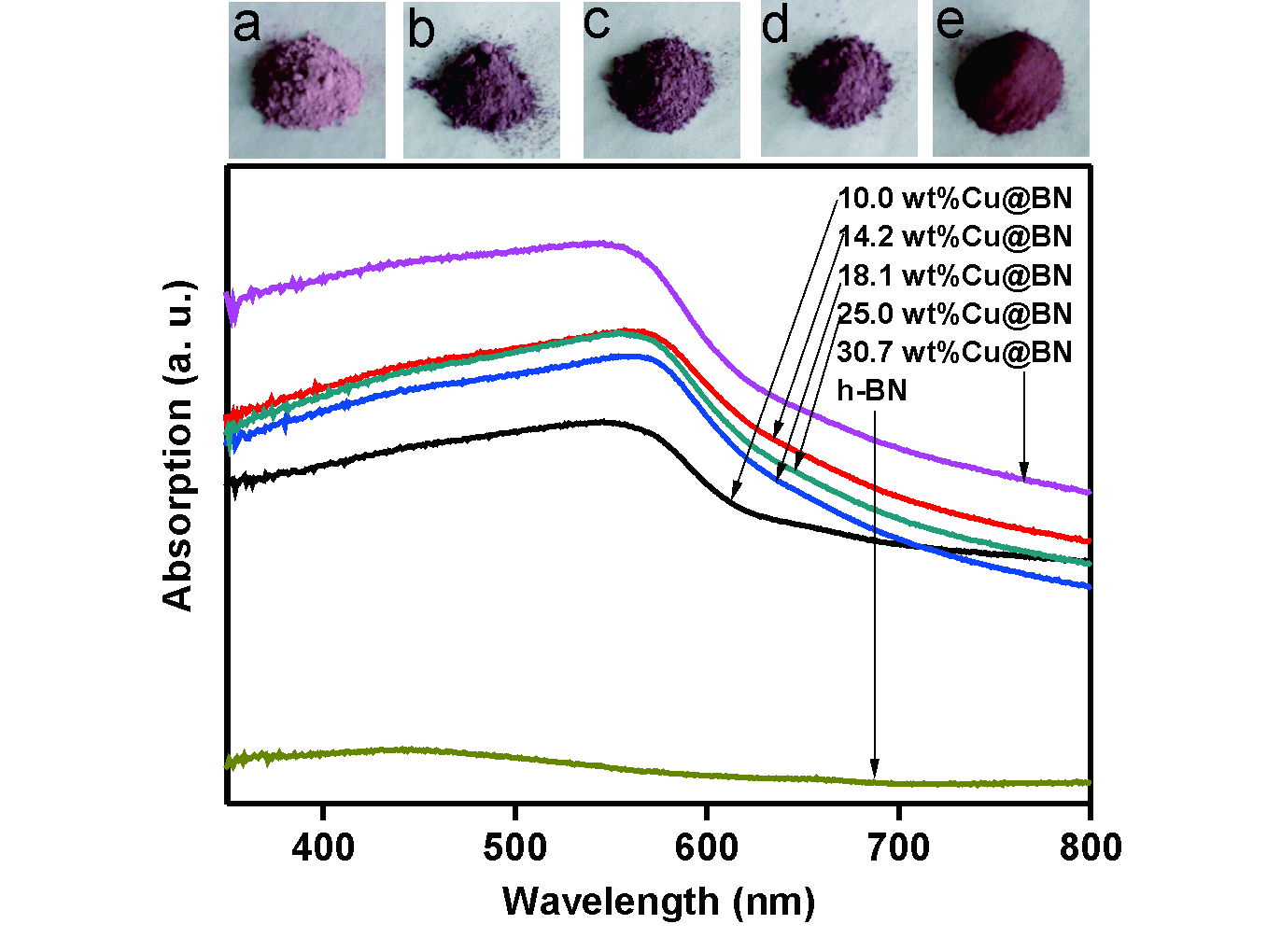


**Figure S2.** DRS of *h*-BN and x-Cu@*h*-BN. Top panel: the photos of (a) 10.0 wt% Cu@*h*-BN, (b) 14.2 wt% Cu@*h*-BN, (c) 18.1 wt% Cu@*h*-BN, (d) 25.0 wt% Cu@*h*-BN, (e) 30.7 wt% Cu@*h*-BN, respectively.

**Figure S3.** Raman spectra of *h*-BN, 25.0 wt% Cu@*h*-BN and 30.7 wt %Cu@ *h-*BN.

Figure S4. (a) XPS spectra of the as-obtained 25.0 wt% Cu@*h*-BN powder. (b) Cu LMM auger electron spectra of 25.0 wt% Cu@*h*-BN.

Figure S5. (a) DTA survey of the 25.0 wt% Cu@*h*-BN samples :AP = 1:99, 2:98, 3:97, 5:95 and 10:90 wt/wt, and (b) heat release during the exothermic process occurred in (a).

Figure S6. DTA analyses for the freshly prepared Cu@*h*-BN (25.0 wt%) and the same sample stored in air for 3 months.

Figure S7. XRD of 25.0 wt% Cu@*h*-BN after reaction.

Table S1. Cu 2p XPS and Cu Auger parameters of 25.0 wt% Cu@*h*-BN.

| **Sample** | **BE Cu2p_3/2_(eV)** | **KE of**  **Cu LMM** | **ɑ-Cu** |
| --- | --- | --- | --- |
| **25.0 wt% Cu@*h*-BN** | 933.9 | 918.0 | 1851.9 |
| **Cu(metal)^a^** | 932.8 | 918.6 | 1851.4 |
| **Cu_2_O^a^** | 932.8 | 916.5 | 1849.3 |
| **CuO^a^** | 934.6 | 917.1 | 1851.7 |
